# Point-of-Sale Tobacco Advertising and Display Bans: Policy Evaluation Study in Five Russian Cities

**DOI:** 10.2196/publichealth.6069

**Published:** 2017-08-15

**Authors:** Ryan David Kennedy, Ashley Grant, Mark Spires, Joanna E Cohen

**Affiliations:** ^1^ Institute for Global Tobacco Control Department of Health, Behavior & Society Johns Hopkins Bloomberg School of Public Health Baltimore, MD United States

**Keywords:** tobacco, marketing, public health, public policy, evaluation, Russia

## Abstract

**Background:**

The tobacco industry uses point-of-sale (POS) advertising, promotion, and product display to increase consumption of its products among current users, to attract new consumers, and to encourage former customers to resume tobacco use. As part of a comprehensive tobacco control effort, Russia—having one of the highest tobacco use prevalence rates in the world—enacted legislation that banned tobacco POS advertising, effective November 15, 2013, and banned the display of tobacco and the sale of cigarettes in kiosks, effective June 1, 2014.

**Objective:**

The objective of the study was to evaluate the implementation of the national law by assessing the state of POS advertising, promotion, and product display, and sales in kiosks across Russia.

**Methods:**

Two waves of observations were conducted to measure compliance with the POS restrictions: wave 1 took place in April-May 2014 after the advertising ban was in effect and again in August-September 2014 after the display ban and elimination of tobacco sales in kiosks came into effect. Observations were conducted by local trained staff that traveled to 5 populous cities in different regions of Russia (Moscow, St. Petersburg, Kazan, Ekaterinburg, and Novosibirsk). Staff followed a published POS evaluation protocol and used mobile phones to collect data. Observations were conducted in a roughly equal number of supermarket chains, convenience stores, and kiosks. Observed items included advertising at POS, product displays, and cigarette sales in kiosks.

**Results:**

Observations were made in 780 venues in wave 1 and in 779 revisited venues in wave 2. In wave 1, approximately a third of supermarkets and convenience stores (34.2%, 184/538) were advertising cigarettes using light boxes, and over half of observed venues (54.3%, 292/538) had signage such as banners or shelf liners that used colors or images related to cigarette brands. Product displays were common in wave 1. In wave 2, compliance with advertising restrictions was very good: there were virtually no light boxes (1.0%, 5/489); banners or shelf liners were observed in 30.5% (149/489) of supermarkets/convenience stores; approximately 7.4% (36/489) of venues were still displaying products in a powerwall. In wave 2, 41.3% (100/242) of kiosks continued to sell tobacco.

**Conclusions:**

Russia’s compliance with POS bans was excellent. Remaining compliance issues are largely with the use of cigarette brand colors or images used in banners or shelf liners; this type of infraction is more difficult to enforce as inspectors need to be deeply familiar with tobacco industry products and marketing practices. A sizable proportion of kiosks continue to sell tobacco post restrictions.

## Introduction

### Tobacco Use in Russia

Tobacco use is a worldwide problem exacerbated by a global tobacco industry that works to promote and sell a product that kills nearly half of its long-term users [1]. The health burden of tobacco use is borne heavier in certain countries and regions due in part to higher prevalence of use. One of the countries most affected in the world is the Russian Federation (Russia). The World Health Organization (WHO) estimates that just over 60% of adult males and almost 22% of adult females smoke cigarettes (a total of approximately 43.9 million adults) [2]. Every year, it is estimated that 400,000 Russians are killed by tobacco-caused disease [2].

Russia represents the world’s second largest tobacco market by volume of sales, worth an estimated US $28 billion in 2014 [2,3]. It is well documented that the tobacco industry works to increase sales of its products using a variety of tobacco advertising, promotion, and sponsorship (TAPS) activities [4,5], and that these activities increase tobacco consumption among current users, attract new consumers, and encourage former customers to resume tobacco use [4-7]. The tobacco industry spends tens of billions of US dollars globally each year on TAPS [2,5]. In Russia, it is estimated that the tobacco industry invests approximately US $1 billion annually on TAPS [8,9].

### Tobacco Promotion in Russia

Before the 1970s, consistent with a centralized economy, not much commercial advertising took place in Russia [10]. This began to change in the 1970s when Western-made cigarette brands were introduced to Russia. In 1980, the Soviet government adopted a regulation that banned the advertising of cigarettes in mass media and on outdoor billboards; the regulation was largely followed until the end of the decade [10,11]. However, by the mid-1990s, tobacco advertising in Russia was ubiquitous; ads were on television and radio, and cigarette products were promoted on billboards, in public transit spaces, and at point-of-sale (POS), including brightly colored kiosks that replaced “gloomy grey tobacco kiosks” [10]. It was noted that in the 1990s, the most prominent product being sold in kiosks was cigarettes [12]. In the mid-1990s, foreign cigarette companies were reported as being the largest advertisers on television and radio—accounting for up to 40% of Russia’s national advertising [13].

### Tobacco Control in Russia

The Russian Ministry of Health was actively working to develop tobacco prevention and control programs during the 1990s [9]. Earlier versions of the Federal Law No. 87-FZ of July 10, 2001, on the “Imposition of Restrictions on Tobacco Smoking” included limitations on tobacco advertising; however, these limitations were removed from the actual law that was passed [9]. A federal law regulating all commercial advertising was passed in the Duma in 2006 (Federal Law No. 38-FZ ); Article 23 of the advertising law provided key provisions governing the advertising of tobacco products, including banning tobacco advertising in TV and radio programs and in videos and movies. The law further banned advertising in printed publications intended for minors, and newspapers and magazines could not print tobacco advertisements on the first and last pages [14]. Although this law represented significant progress for tobacco control, the tobacco industry continued to sponsor events, and advertise and promote its products in public spaces and on billboards, and, importantly, at POS [9].

In 2003, to address the global problem of tobacco use, an international treaty was negotiated under the auspices of the World Health Organization (WHO): the Framework Convention on Tobacco Control (FCTC) [15]. The FCTC outlines effective policy responses and implementation guidelines to support tobacco control including measures against TAPS [4]. Comprehensive bans on TAPS have proven to be the most effective in reducing tobacco consumption [16-18], particularly product displays at POS [19-21]. There is evidence that although a country restricts tobacco product advertising in media, tobacco companies will increase their marketing efforts through other channels including POS promotions [22]. The WHO reported in 2013 that 50% of adult Russians notice POS promotions for tobacco products [23], suggesting that this was an important marketing strategy for the tobacco industry.

**Figure 1:**
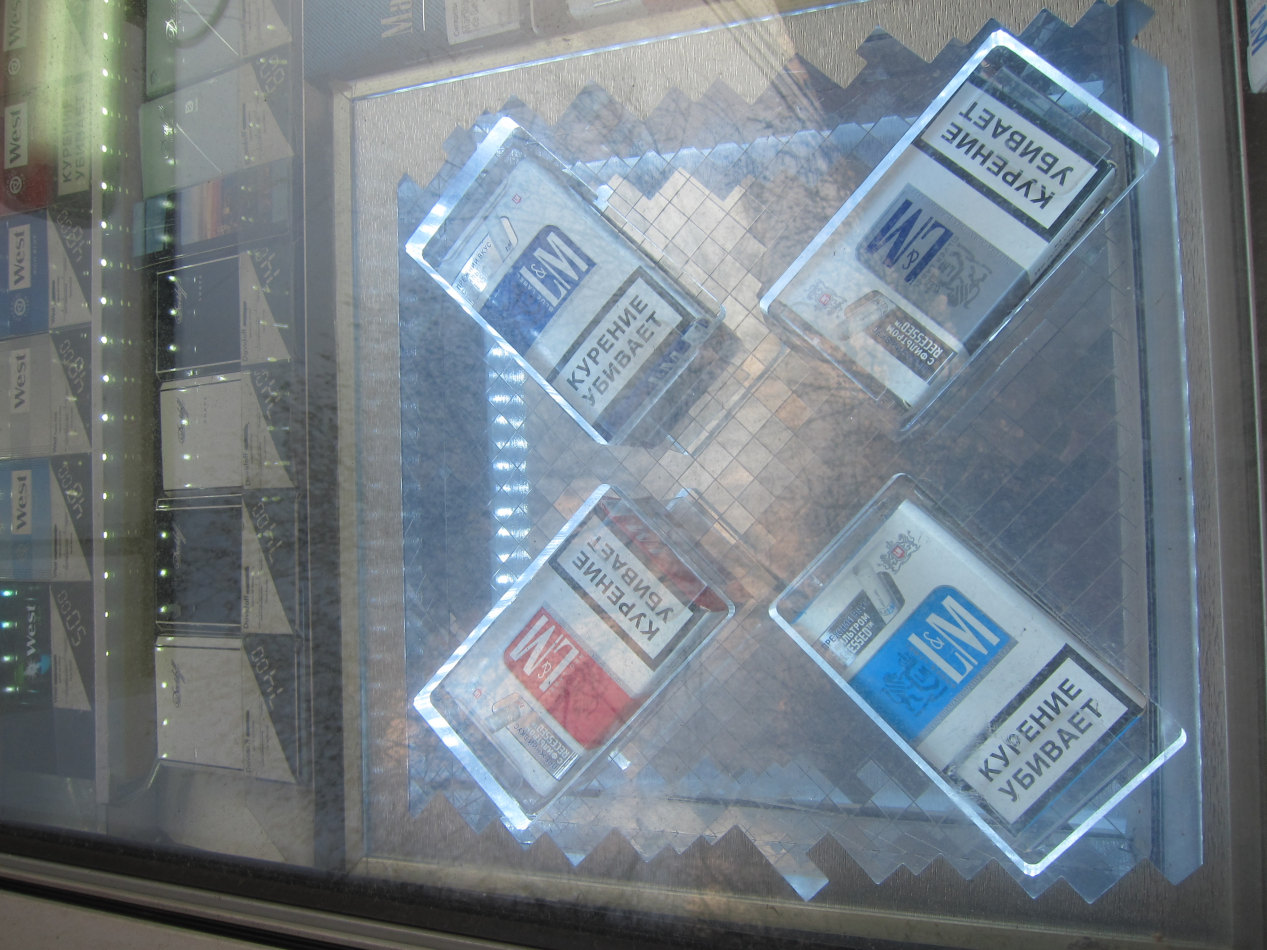
Cigarette packs displayed in a lightbox, Moscow, April 17, 2014. (photo credit: Ashley Grant).

Russia acceded to the FCTC on 3 June, 2008, and, on February 23, 2013, passed the Federal Law N 15-FZ, “Protecting the Health of Citizens From the Effects of Second Hand Tobacco Smoke and the Consequences of Tobacco Consumption” (tobacco control law) [24]. The tobacco control law was implemented in two phases. The first phase, outlined in Article 16, bans all forms of tobacco advertising, promotion, and sponsorship, and came into effect on November 15, 2013. This included banning marketing at POS such as promotional signage, price discounts, and free product giveaways. The second phase, outlined in Article 19, prohibits the display of tobacco products at trade sites and regulates retail product listings; these restrictions came into effect on June 1, 2014. The tobacco industry quickly identified a possible loophole during the implementation of Article 16, determining that enhanced forms of product displays could be considered compliant with advertising restrictions. This perceived loophole was exploited by employing tactics such as light boxes (see Figure 1) and enlarged cigarette packages. There was no legal decision made in Russia to determine whether such product displays could be classified as advertising or promotion or whether they would be considered product display. Article 19 also restricted the sale of tobacco to only stores and pavilions, which were defined in part using various physical characteristics including having a door for customers to enter. Kiosks, which generally sell items through a window, do not match the definition of a store or a pavilion and therefore were not permitted to sell tobacco after June 1, 2014. The Federal Department of Health has argued that kiosk vendors often sold cigarettes to minors [25]. As described earlier, kiosks also prominently display cigarettes in their windows and often include bright colorful advertisements for cigarette products.

This two-phase policy implementation approach presents an important opportunity to understand how retailers achieve compliance with different types of advertising, promotion, and display restrictions and a ban on sales in a specific vendor type. Policy evaluations are useful to inform policy implementation and enforcement and to identify any policy development needed to close loopholes that undermine the spirit of the legislation.

This aim of this study is to use a policy design to evaluate the implementation and compliance of the national law, before and after it was enacted, by assessing the state of POS advertising, promotion, and product display, and sales in kiosks across Russia. This study design captured industry activities between the implementation of product advertising and promotion and product displays, which provides insight into the tobacco industry’s marketing efforts and optimal policy design. Finally, the study measured the sale of other non-cigarette products including alcohol, e-cigarettes, and gasoline to understand, in part, if this tobacco control legislation is associated with unintended consequences such as a change in the availability of other consumer products with potential public health impacts.

## Methods

### Groups Involved in the Study

This work was conducted by researchers based at the Institute for Global Tobacco (IGTC) at Johns Hopkins Bloomberg School of Public Health in Baltimore, Maryland (USA) referred to in this paper as the investigator team. IGTC worked closely with partners of the Campaign for Tobacco Free Kids, an international public health nongovernmental organization based at Washington, DC (USA), which includes policy experts and a legal team. IGTC also partnered with Russian tobacco control experts based in Moscow, who worked as data collectors. These experts were familiar with the tobacco control law and industry marketing practices.

This study was observational in nature; it did not include human subjects and therefore did not require approval from an institutional review board.

### Study Overview

The study details are described in detail below. As a summary, the compliance measures relevant in each wave of data collection are detailed in Table 1, including the dates of data collection and details of the tobacco control law.

**Table 1:** Study overview.

Study domains	Wave 1 Data collected April-May 2014	Wave 2 Data collected Aug-Sept 2014
Tobacco control law details	Article 16, bans all forms of tobacco advertising, promotion and sponsorship, Restrictions entered into effect on November 15, 2013.	Article 19, prohibits the display of tobacco products at trade sites and regulates retail product listings; Restrictions entered into effect on June 1, 2014.
POS (point-of-sale) compliance	All cigarette promotions and advertisements are banned including: Use of signs/ posters/ banners/ shelf liners/ backgrounds (not in light box) Use of light boxes Use of enlarged packs Promotional discounts Sale or distribution of non-tobacco products with a tobacco brand name Signage or brand representative offering gifts (free or with purchase) Free product distribution	All cigarette product displays are banned including: Cigarette pack/product display visible from street (kiosk or storefront window) Cigarette pack/product display in cashier zone Cigarette pack/product display on power wall Cigarette pack/product display in other locations Product sales of cigarettes only permitted in stores or pavilions (not permitted to be sold in Kiosks); Product list required to be available upon request.
Other products sold or displayed (unintentional consequences of the law)	Gasoline, e-cigarettes, alcohol, smokeless tobacco	Gasoline, e-cigarettes, alcohol, smokeless tobacco

The protocol used in this study is detailed extensively elsewhere [26], but in brief, the study was conducted in the cities of Moscow, St. Petersburg, Novosibirsk, Ekaterinburg, and Kazan. These cities were selected for data collection based on their population size and geographical dispersion. Each of the 5 cities is located in separate and distinct federal subjects (constituent members of the federation) and is among the top 10 most populous cities in Russia. POS venues included in the study were located in neighborhoods with varying property values across each city; roughly equal number of observations were conducted in neighborhoods with above-average, average, and below-average housing value (used as a proxy for socioeconomic status). The Russian based data collectors acquired a near-comprehensive list of supermarkets and their addresses, but did not have access to comprehensive lists of other tobacco retailers. POS venues included in the study were identified by randomly selecting supermarkets where data collectors began recording observations, and by using a walking protocol to identify nearby convenience stores and kiosks. Data collectors were trained to follow the walking protocol (which was designed to be sufficiently random while also expedient) to identify POS venues and to collect observational data by completing an observation checklist. The observation checklist was developed following a thorough review of the tobacco control law and was reviewed by in-country partners including a public health lawyer.

Four data collectors from Russia were trained over 3 days in Moscow in April 2014 and retrained for 2 days before wave 2 in August 2014. Initial training included an introduction to the tobacco control law and different POS marketing or product display practices. The data collectors spent 2 supervised days in Moscow practicing data collection including how to identify POS venues following the walking protocol, how to use the mobile technology, and how to conduct the observations in retail settings. Initially, data collectors practiced conducting observations in pairs under the direct supervision of the investigator team. Each pair of data collectors visited a minimum of 6 POS venues in Moscow to practice conducting observations and uploading data. Then a set of 8-10 POS venues were double-coded by data collectors to ensure consistent and reliable observations. The field team experienced minimal differences in observations. In wave 2, similar practice field work was conducted to reacquaint the data collectors to the mobile technology. Venues were revisited in wave 2, so no walking protocol was needed; photos taken and GPS (Global Positioning System) coordinates collected in wave 1 were used by data collectors to help identify venue locations in wave 2.

Data collectors used mobile phones equipped with a customizable mobile data collection software app to complete observations and, when possible, took digital photos of observed marketing including signage and product displays [26]. Data were uploaded in real time when the phones were connected to a cellular network. Data included the aforementioned specific instrument observations and photos as well as metadata (such as time stamp, GPS-based location coordinates, and device identification number). Data collectors primarily relied on the cellular network, but did occasionally use Wi-Fi capabilities to enhance the accuracy of study site geolocation. The real-time data upload allowed the investigator team in Baltimore, Maryland, to oversee the field work.

Data collectors were required to take photos of the POS entrance, which was particularly important to ensure that the wave 2 data collection occurred at the same venue. The data collectors were also asked to take photos of product displays and advertising or promotional activities. However, these were optional because data collectors were occasionally reprimanded by store clerks or security guards; therefore, data collectors prioritized collecting observational data and took photos when it was possible. Data collectors could also add in specific notes to each POS observation, and were required to complete daily field reports highlighting any data collection issues or questions along with the number of POS venues visited that day. The Baltimore-based investigator team could function as a remote field supervisor and review uploaded data in real time, and in some cases was able to respond to any questions related to observation classifications or the walking protocol within 24 hours. Time differences made immediate response challenging, but data sharing occurred automatically without the need to wait for data collectors to share files. Members of the investigator team could check for data accuracy or possible inconsistencies such as GPS coordinates corresponding with POS location, or review images collected in POS environments and ensure the details in the images matched the recorded observations.

Data were collected at two points in time: wave 1 was conducted in April-May of 2014 (5 months after the tobacco control law banned all forms of advertising and promotion), and wave 2 was conducted in August-September 2014, 3 months after Article 19 (the second phase of Russia’s tobacco control law) was implemented, which banned all product displays and sales of tobacco products in kiosks. Before wave 2, data collectors underwent similar training and pilot testing as in wave 1. The data collectors in wave 2 of this study included 2 staff members who participated in wave 1 and 2 staff members who were new to the study.

Physical details about kiosks (the presence of a door for customers to enter or exit) were also collected in order to distinguish them from stores and pavilions.

### Sample

**Figure 2:**
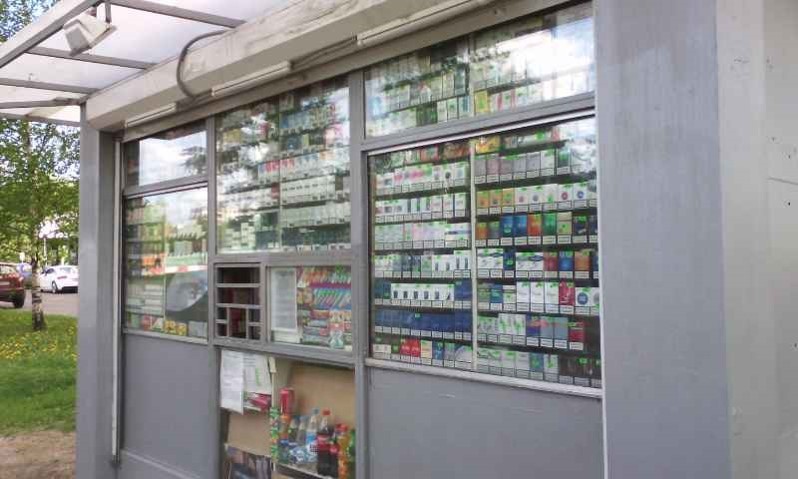
Kiosk selling tobacco in Moscow, April 17, 2014 (photo credit, Ashley Grant).

The study measured compliance with the tobacco control law in different types of retail settings, including: (1) supermarket chains, (2) independently owned markets/convenience stores (including gas stations), and (3) kiosks. These POS types were selected for inclusion based on their prominence as tobacco retailers in Russia. Kiosks were also included because Article 19 permits the sale of tobacco only in stores and pavilions. See Figure 2 for an image of a kiosk in Moscow with cigarette packs on display before the product display ban took effect.

The data collectors had a goal to visit 810 POS in wave 1—162 POS in each city (54 in each high, medium, and low property value neighborhoods, with 18 venues from each type of retail setting). A goal was more suitable than a quota, because the availability of some types of retailers is not uniformly distributed in each neighborhood. A list of supermarkets in each of the 5 cities was created using a variety of mapping tools including 2GIS, Google Maps, and Yandex. Data collectors would begin data collection at a supermarket, exit the store, and follow a prescribed walking protocol to identify a convenience store and a kiosk (for which there were no lists and addresses available). The protocol had a provision that if no convenience store or kiosk was found after 30 minutes of walking, data collectors could proceed to the next supermarket on their list [26]. In wave 2, data collectors returned to the same venues to repeat observations.

### Observations

In wave 1, data collectors visited retail venues and noted (yes/no) *cigarette product advertisements* including signs, posters, banners, shelf liners or backgrounds, a light box, and the presence of oversized cigarette packs. Tobacco companies provide some retailers with signage or display cases that use tobacco brand colors or images that can convey the brand. Data collectors also noted (yes/no) *cigarette product promotions* including coupons, discounts or vouchers, brand stretching, gifts with purchase, and the presence of brand representatives and free product giveaway promotions. Data collectors noted the brands of cigarettes being promoted if any of the above tactics were being used. Data collectors also recorded the presence of *cigarette product displays*. Data collectors noted (yes/no) if cigarettes were visible from the street, in the cashier zone, on a powerwall, and in any another area of the retail venue (area recorded).

Additional consumer product displays, not regulated through the national tobacco control law, were also noted (yes/no) including displays for e-cigarettes and smokeless tobacco and the presence of candy or sweet snacks on display in the cashier zone. Furthermore, the sale (yes/no) of alcohol and e-cigarettes was also noted. If e-cigarettes were not on display, data collectors asked the staff at the venue if these products were available.

In wave 2, data collectors noted any changes in the status of the POS, for example, whether the business had closed or ceased selling cigarettes. If a POS continued to sell cigarettes, in lieu of product displays, the retailer was allowed by the tobacco control law to have a list of tobacco products sold, the text of which is in letters of the same size in black font against a white background and which is composed in alphabetical order, with indication of the price of tobacco products sold without the use of any graphics and images [25]. Data collectors recorded if the POS had a *product list* (yes/no), and if it was in compliance (yes/no) with the law’s requirements including being printed on plain white paper with text in a black font.

In both waves 1 and 2, data collectors could also record general comments or notes about a venue. At the end of each day, data collectors completed a log summarizing the number and type of venues they visited and any issues with data collection.

### Data Quality Checks

Data collected in Russia were reviewed, generally within hours, by the Baltimore-based investigator team who routinely checked time stamps and GPS locations to ensure data collectors were in the correct locations and that their daily logs matched the uploaded files.

### Analysis

Observations are reported as proportions of noncompliance with the tobacco control law; analysis was conducted by the lead author (RDK) and coauthor (AG), members of the Baltimore-based investigator team. Analysis was conducted using SPSS statistical software version 23.0 (IBM Corp). Some of the recorded observations, including brand of cigarettes advertised or on display, were manually counted. Additional observation notes were reviewed and reported.

## Results

### Sample

The study team conducted observations and recorded data at a total of 780 POS in wave 1; the number of POS observed by city, relative property value zone, and retail type are detailed in Table 1. During wave 2 of data collection, data collectors revisited 779 POS venues (one location, a kiosk, could not be located); data collected and uploaded successfully in wave 2 (n=720) are also detailed in Table 2.

**Table 2:** Cigarette POS (point-of-sale) venues where observations took place during wave 1 and wave 2.

City	Supermarket chains		Independent market or convenience store		Kiosk		Total per city	
	Wave 1	Wave 2	Wave 1	Wave 2	Wave 1	Wave 2	Wave 1	Wave 2
Moscow	59	54	54	50	54	46	167	150
St. Petersburg	53	52	54	51	49	45	156	148
Novosibirsk	55	53	53	50	54	49	162	152
Ekaterinburg	53	54	54	49	55	45	162	148
Kazan	51	50	52	49	30	23	133	122
Total per venue type	271	263	267	249	242	208	780	720

In wave 1, sample goals were achieved or nearly achieved in each city. In wave 2, one venue could not be relocated and thus was not observed. Data from 7 venues were not successfully uploaded due to failure of mobile devices. When visited, 52 POS venues were closed, and observers determined the retail venues were unlikely to reopen in the near future, and therefore observations were not possible. The majority of venues that were closed were kiosks (63%, n=33), followed by convenience/stores (29%, n=15) and supermarket chains (8%, n=4). In total, 720 venues were revisited by data collectors in wave 2 and observations were conducted.

Of the 720 POS venues visited in wave 2, it was determined that 589 were still selling cigarettes. Of the 131 venues that had stopped selling cigarettes, the majority were kiosks (82.4%, n=108), followed by supermarket chains (10.7%, n=14) and independent convenience stores (6.9%, n=9).

### Observations

Observed noncompliance with the tobacco control law in supermarkets and independent convenience stores is detailed in Table 3. Observations conducted at kiosks are reported in Table 4; kiosks are reported separately because in wave 2 no kiosk was permitted to sell cigarettes and was therefore inherently noncompliant.

During wave 1, all product promotions and advertisements were banned; during wave 2, all product display activities were also banned and sales of cigarettes were limited to stores and pavilions (ie, banned at kiosks). Cigarettes displayed in light boxes and enlarged packs are presented in Tables 3 and 4 as product promotions and advertisements; however, as described earlier, the industry preferred to consider these marketing tactics as product displays.

**Table 3:** Noncompliance with POS (point-of-sale) restrictions at supermarkets and convenience stores.

Observation item		Wave 1 (N=538), n (%)	Wave 2 (N=489)
**Product promotions and advertisements**			
	ANY cigarette promotion or advertisement	367 (68.2)	150 (30.7)
	Use of signs/posters/banners/shelf liners/backgrounds (not in light box)	292 (54.3)	149 (30.5)
	Use of light boxes	184 (34.2)	5 (1.0)
	Use of enlarged packs	31 (5.8)	1 (0.2)
	Promotional discounts	9 (1.7)	0
	Sale or distribution of non-tobacco products with a tobacco brand name	17 (3.2)	Data not available
	Signage or brand representative offering gifts (free or with purchase)	22 (4.1)	0
	Free product distribution	21 (3.9)	2 (0.4)
**Product display**			
	ANY display of cigarette pack/product	510 (94.8)	109 (22.3)
	Cigarette pack/product display visible from street (storefront window)	18 (3.3)	2 (0.4)
	Cigarette pack/product display in cashier zone	492 (91.4)	104 (21.3)
	Cigarette pack/product display on a powerwall	181 (33.6)	36 (7.4)
	Cigarette pack/product display in other locations	30 (5.6)	6 (1.2)
	Smokeless tobacco products on display anywhere in store	23 (4.3)	1 (0.2)
	Cigarette product list—noncompliant or no list	N/A	259 (53.0)

**Table 4:** POS (point-of-sale) promotions and product displays observed at kiosks.

Observation item		Wave 1 (n=242)	Wave 2 Open and selling tobacco (n=100)
Product promotions and advertisements			
Venue has a door		52 (21.5)	67 (67.0)
**ANY cigarette promotion or advertisement**			12 (12.0)
	Use of signs/ posters/banners/shelf liners/backgrounds (not in light box)	121 (50.0)	12 (12.0)
	Use of light boxes	67 (27.7)	0
	Use of enlarged packs	11 (4.5)	0
	Promotional discounts	2 (0.8)	0
	Sale or distribution of non-tobacco products with a tobacco brand name	6 (2.5)	Data not available
	Signage or brand representative offering gifts (free or with purchase)	6 (2.5)	0
	Free product distribution	4 (1.7)	0
Product display			
**ANY display of cigarette pack/product**		242 (100.0)	24 (24.0)
	Cigarette pack/product display visible from street (kiosk or storefront window)	206 (85.1)	0
	Cigarette pack/product display in cashier zone	234 (96.7)	22 (22.0)
	Cigarette pack/product display on a powerwall	90 (37.2)	9 (9.0)
	Cigarette pack/product display in other locations	4 (1.6)	1 (1.0)
	Smokeless tobacco products on display anywhere in store	20 (8.3)	2 (2.0)
	Cigarette product list—noncompliant or no list	N/A^a^	78 (78.0)

^a^N/A: not applicable.

In wave 1, the overwhelming majority of POS venues were compliant with restrictions related to price discounts, sales of non-cigarette products with cigarette branding (brand stretching), signage, or a brand representative present offering gifts (free or with purchase) or distributing free product. Approximately 5.8% (31/538) of supermarkets and convenience stores and 4.5%, (11/242) of kiosks had enlarged packs on display; brands of oversized packs included Marlboro, Lucky Strike, Bond, Chesterfield, and Kent. Approximately one-third (34.2%, 184/538) of supermarkets and convenience stores and about a quarter of kiosks (27.7%, 67/242) had a light box advertising or displaying a brand of cigarette. The most offending brand by far was Kent (present in 16.4% of all POS venues (128/780), as well as several other brands including Camel, Chesterfield, Davidoff, Marlboro, Parliament, and Winston.

The most common noncompliance issue in wave 1, observed in over half the POS venues, was the presence of signs, shelf liners, or backgrounds that used colors or symbols from a tobacco brand.

Product displays were not banned at the time of wave 1 data collection, and the vast majority of all POS venues had products visible in the cashier zone including 91.4% (492/538) of supermarkets and convenience stores and 96.7% (234/242) of kiosks. Products were visible from the street for most kiosks (85.1%, 206/242), compared with only 3.3% (18/538) of supermarkets and convenience stores.

In wave 2, cigarette advertising and promotions decreased. The use of light boxes and oversized packs had largely ceased: approximately 1% of POS venues had light boxes (5/489) and only one POS had an enlarged pack (Marlboro). Free product distribution was observed in 2 POS venues. Due to a glitch in the mobile data collection software, observations of the sale or distribution of non-tobacco products with a tobacco brand name were lost during the upload of data, although very few supermarkets or convenience stores (3.2%, 17/538) and fewer kiosks (2.5%, 6/242) were noncompliant in wave 1, and observer photos and notes did not include evidence of this brand stretching in wave 2. Almost one-third of POS venues (30.5%,149/489) continued to have signs/posters/shelf liners, or backgrounds that used colors or symbols from a tobacco brand. The brand colors used included Bond, Chesterfield, Kent, Marlboro, Parliament, and Rothmans.

In wave 2, 2 supermarket/convenience stores POS had a pack visible from the street. Some of these venues continued to have packs visible on a powerwall (7.4%, 36/489) or in the cashier zone (21.3%,104/489); it was noted by data collectors that some products were visible in the cashier zone or on a powerwall because staff had improperly or incompletely covered cigarette packs with curtains or because the curtains were not sufficiently opaque. One data collector noted during a visit to a supermarket in Ekaterinburg that a cashier reminded their colleague on two occasions to close the door to a cupboard that was displaying cigarettes.

In wave 2, it was observed that 100 kiosks remained open and sold tobacco. Data collectors observed that the majority of these venues, two-thirds (67/100), had a door, and approximately half of these venues (29/67) had added the door since wave 1.

In wave 2, more than half of supermarkets and convenience stores and more than three-quarters of kiosks selling tobacco neither had a cigarette product list nor had a list that was noncompliant because it was not printed on the correct style of paper or with the correct font or other design issues.

In wave 1 and wave 2, almost all venues displayed candy or sweets in the cashier zone (approximately 95% in both waves). In wave 1, smokeless tobacco was on display in the cashier area of some supermarkets and convenience stores (4.3%, 23/538); however, in wave 2, only one convenience store displayed smokeless tobacco. In wave 1, e-cigarettes were on display in about a quarter of venues (26.3%, 205/780) and for sale in 31.0% of venues (242/780). Over 90% of supermarkets and convenience stores sold alcohol in each wave. In wave 1, 14.0% (34/242) of kiosks sold alcohol; of the kiosks still selling tobacco in wave 2, 25.0% (25/100) also sold alcohol.

Compliance with product display restrictions in wave 2 differed across cities. In Ekaterinburg, approximately half of POS venues (51.2%, 66/129) were noncompliant, compared with 10.7% in Kazan (11/103), 13.8% in Novosibirsk (15/109), 15.0% in St. Petersburg (20/133), and 15.7% in Moscow (18/115).

During data collection in wave 2, recorded observations about the sale or distribution of non-tobacco products with a tobacco brand name were not properly uploaded and those fields were left blank. During wave 1, these forms of promotion were observed in 2.9% (23/780) of venues.

## Discussion

### Policy Implementation

The results of this study demonstrate that the tobacco control law in Russia that banned tobacco advertising, promotion, and product display has been well implemented, with the vast majority of retailers compliant with these restrictions. The multiphase aspect of the POS restrictions demonstrate that the tobacco industry took advantage of some ambiguity in the law, and continued to use tactics such as light boxes and enlarged packs after the implementation of product advertising and promotional restrictions; however, these tactics almost vanished after the product display bans were implemented, pointing to the need for clear and comprehensive policy language. There was a notable difference in compliance between retail venues in Ekaterinburg and the venues in the other 4 cities. The reasons for this are unknown, but do suggest that implementation of the law was not uniform across the country. The study also revealed that there were only minor changes in the display and sale of products including alcohol, e-cigarettes, and candy in the venues studied. It does not appear that kiosks replaced sales of cigarettes, for example, with alcohol or e-cigarettes. The results of this study are similar to other POS studies before and after the ban that found tobacco retailers were almost universally compliant following the implementation of comprehensive laws [20,27,28].

This study also found that the majority of kiosks achieved compliance with the tobacco control law by no longer selling tobacco (51.9%, n=108). About 1 in 7 kiosks were closed at the time of wave 2 data collection, although some supermarkets and convenience stores were also closed between waves, suggesting that there is a rate of retail closure independent of this legislation. The tobacco control law requires tobacco retailers to provide a product list, and the law sets out very specific criteria for that list in terms of font size and paper color. Many POS venues were not compliant with this aspect of the law; however, the impact on public health from this aspect of noncompliance is likely negligible.

In wave 2, some POS venues (less than a third) had signage or cabinets (formerly display cases) that included brand colors or images that are associated with cigarette brands. This type of infraction is difficult to enforce as inspectors need to be deeply familiar with tobacco industry products and marketing. Light boxes and, to a lesser extent, oversized cigarette packs were common after the implementation of the national ban on tobacco promotion and advertising; however, virtually none were observed following the product display ban.

Approximately a quarter of venues had cigarettes visible in the cashier zone. This can be partially explained by cupboard doors and curtains not being fully closed. Display bans that require tobacco products be kept under a counter do not have these staff-related compliance issues because the products are more likely to be out of view of the customers.

Kiosks were a common place to purchase cigarettes before the implementation of Article 19 of the tobacco control law, and almost all kiosks included in this study had cigarette packs visible from their storefront. Kiosks arguably were not just a place to purchase cigarettes, but would have greatly impacted the visual landscape of the cities included in this study. It is uncommon globally for a specific type of retailer to be banned from selling cigarettes. Although some supermarkets and convenience stores closed between the waves, a greater proportion of kiosks were closed at the time of wave 2 data collection. Presumably, this can be explained in part by the fact that some kiosks in our sample likely relied primarily on cigarette sales and would clearly have been impacted deeply by the tobacco control law. At the time of wave 2, it was unclear if some kiosks were closed permanently or if they were closed while reconfiguring to sell different products. Some data collector notes suggested that some kiosks were now selling shoes or ice cream although our data collection methods did not allow us to measure changes in product offerings. Despite some possible closures, many kiosks remained open and achieved compliance with the law by ceasing to sell cigarettes. The facades of these outlets were transformed as a direct result of the ban on the display of cigarette packs, which were, when collectively displayed in kiosk windows, essentially acting as large advertisement for the products. As noted, most of the kiosks still selling cigarettes had a door (and many added a door between waves). Presumably kiosk owners or managers had added a door in an effort to achieve compliance with the law; however, it is unclear if this simple physical change would be sufficient to reclassify the POS venue as a store or a pavilion.

There was little change between the waves in the proportion of POS venues selling e-cigarettes, a class of products that was excluded from the definition of tobacco products in the tobacco control law. Moving forward, it will be important to monitor e-cigarette displays and advertising in Russia. In wave 2, the proportion of kiosks selling alcohol was slightly greater than in wave 1. It will also be important to understand if kiosks have transitioned from the sale of cigarettes to the sale of alcohol, as this may trade one public health issue for another.

### Lessons and Opportunities for Future Mobile Data Collection

Russia is a large country, both in geography and in population. These factors can present challenges for tobacco control policy implementation and evaluation. The mobile data collection method followed by this study was effective and presented few problems. Observational data, including photos as well as metadata such as including the time when data were logged, and the GPS coordinates worked well and permitted the research team in the United States to monitor work as it was conducted, ensuring that the team’s data were collected in the proper locations and that observational data corresponded with the image content. The field team did experience an issue with uploading data from 7 POS venues, and it was determined that this was due to a network connectivity issue. In wave 2, one variable related to the sale or promotion of non-tobacco products with a tobacco brand name (brand extension) was not uploaded properly, and that variable was not reported. Finally, there was one location (a kiosk) that could not be found in wave 2—one probable explanation was that the kiosk was physically removed and therefore it was impossible to find. These issues, related to methods, were small with respect to the use of these novel protocols and demonstrate that this method should be considered by other jurisdictions to evaluate similar policies related to POS policy restrictions.

The protocol, designed for expediency as well as rigor, did not include a step where a subset of venues was visited by multiple data collectors. This decision results in limitations to the study. First, data collectors may have missed or incorrectly recorded certain important observations. Using double-coding may have reduced the likelihood of something being missed. Second, without double-coding venues, there is less certainty that data collectors did not fraudulently fabricate observations. There are several reasons to believe that these are not deep concerns, including the fact that data collectors were observing the presence of promotional materials such as posters, which are, by nature, visible. Second, the data collectors were aware that data were being checked as they were uploaded. The metadata include time and location; their in-store observations, however, could have been fabricated. The protocol required data collectors to take pictures where possible, inside venues, which allows for some secondary checks to ensure accuracy with data collection. Although most POS venues included at least one photo, not all photos were taken with sufficient resolution or appropriate in-frame content to confirm all data collection details. It is suggested that future studies consider including a subset of venues to be visited by multiple data collectors to improve the rigor of findings.

### Implications for Public Health and Policy

This study found higher compliance in wave 2, when product displays were banned in addition to product advertisement and promotion; in particular, there was a decrease in product displays including light boxes, which were present at approximately one-third of venues during wave 1 but almost nonexistent during wave 2. This highlights the benefit of comprehensive policies where there is little opportunity for ambiguity in the law.

Comprehensive policy evaluations provide evidence of policy compliance and can help measure unintended consequences from the policy. This study did not examine implementation strategies related to how the Russian government implemented the tobacco control law provisions regarding tobacco advertising, promotion, and product display. Understanding these approaches and strategies can be helpful for other jurisdictions preparing to implement similar policies. Other researchers have focused on the challenges related to implementing other aspects of the tobacco control law in Russia, including the high demands the law places on Russia’s health care system and the need to establish smoking cessation services to comply with the tobacco control law [9].

The results of this study are important for the preservation of tobacco control policies in Russia and to support the development and implementation of similar policies in other jurisdictions. Globally, there was deep skepticism when jurisdictions such as Ireland and France went smoke-free [29,30]. The results of the present evaluation may support the maintenance of the current ban because tobacco control experts can highlight the success of the policy implementation and highlight that there has not been a significant increase in the sale of other products that present challenges to public health such as alcohol or e-cigarettes. This study’s findings may support the development and implementation of similar policies in other jurisdictions.

## Abbreviations

FCTC: Framework Convention on Tobacco Control
GPS: global positioning system
IGTC: Institute for Global Tobacco Control
POS: point-of-sale
TAPS: tobacco advertising, promotion, and sponsorship
WHO: World Health Organization
